# Screening of the Potential Bioactivities of Pennyroyal (*Mentha pulegium* L.) Essential Oil

**DOI:** 10.3390/antibiotics10101266

**Published:** 2021-10-18

**Authors:** Ângelo Luís, Fernanda Domingues

**Affiliations:** 1 Health Sciences Research Centre (CICS-UBI), University of Beira Interior, Avenida Infante D. Henrique, 6200-506 Covilhã, Portugal; fdomingues@ubi.pt; 2 Pharmaco-Toxicology Laboratory, UBIMedical, University of Beira Interior, Estrada Municipal 506, 6200-284 Covilhã, Portugal; 3 Chemistry Department, Sciences Faculty, University da Beira Interior, Rua Marquês d’Ávila e Bolama, 6201-001 Covilhã, Portugal

**Keywords:** pennyroyal, *Mentha pulegium*, pulegone, antioxidant activity, *Acinetobacter baumannii*, antimicrobial activity, *quorum sensing*, anti-inflammatory

## Abstract

Increasing resistance of pathogens towards conventional antibiotics presents a major threat to public health because it reduces the effectiveness of antibiotic treatment. *Mentha pulegium* L., also known as pennyroyal, is an aromatic herb that belongs to the family Lamiaceae. Its essential oil has been traditionally used in medicine, aromatherapy, and cosmetics. The purpose of this work was to study the chemical composition of a pennyroyal essential oil and to evaluate their bioactivities, specifically, antioxidant, antimicrobial, anti-*quorum sensing*, and anti-inflammatory. A special focus was given to the antibacterial activity of the essential oil against *Acinetobacter baumannii*. The chemical composition of the essential oil was studied by GC-MS/GC-FID. The DPPH free radical scavenging assay and the β-carotene/linoleic acid system were used to evaluate the antioxidant properties. The antimicrobial and anti-*quorum sensing* activities were evaluated by disk diffusion assays and complemented with optical microscopy observations. The results showed that pulegone was the major compound (88.64%) of the pennyroyal essential oil. Regarding the antimicrobial activity, the action against *Acinetobacter baumannii* stands out, which, together with the capacity of the essential oil to inhibit the *quorum sensing* mechanisms, may suggest the use of the pennyroyal essential oil to further develop surface disinfectants for hospitals.

## 1. Introduction

Chemical constituents of plants play an important role in conventional Western medicine. People on all continents have long applied dressings and drank infusions of several indigenous plants to treat or improve their health [1]. In recent years, there has been an increased interest in the bioactive compounds produced by plants, as they are likely to have pharmacological and biotechnological potential [2]. Essential oils (EOs) are complex mixtures of volatile compounds with a strong odor that are synthesized in several plant organs [3]. EOs have numerous therapeutic uses in human medicine owing to their antioxidant [4], antimicrobial [5], and anti-inflammatory [6] properties.

A major threat to public health is caused by the increased resistance of microbial pathogens to conventional antibiotics, reducing their effectiveness and leading to an increase in morbidity and mortality [7]. In recent years, *Acinetobacter baumannii* emerged as one of the most important causes of nosocomial infections related with significant rates of morbidity and mortality [8]. This pathogen is linked to several infectious diseases, including, bacteremia, urinary tract infections, pneumonia, soft tissue, and skin infections [8]. The intrinsic resistance of *A. baumannii* together with its ability to rapidly acquire novel mechanisms of resistance make those infections difficult to treat [8]. Therefore, an urgent demand for new and alternative antimicrobials to treat infections caused by resistant pathogens is needed [7]. Recently, the relationship between *quorum sensing* (QS) mechanisms and the bacterial virulence, associated with the stimulation of the expression of several disease-causing traits, namely, biofilm formation, motility, and virulence factors secretion, was demonstrated [9]. QS is a monitoring mechanism enabling bacteria to collectively decide about the expression of a specific group of genes, contributing to bacterial adhesion, invasion, and resistance [9].

The aromatic herb *Mentha pulegium* L. (European pennyroyal) belongs to the family Lamiaceae. The dry parts of pennyroyal and its EO have been used in traditional medicine (liver and gallbladder ailments, digestive, gout, amenorrhea, increased micturition, colds, skin disorders, and as abortifacient), gastronomy (aromatizing culinary herb), cosmetics, and aromatherapy [10]. In fact, previous reports have shown that *M. pulegium* essential oil exhibited antibacterial activity against several bacterial strains. Moreover, the toxicological profile of pennyroyal has also been subjected to extensive research, and several constituents, particularly terpenoids like pulegone, have been identified as hepatotoxic and abortifacient [10].

The main goal of this work was to study the chemical composition of a Portuguese pennyroyal EO and to evaluate the antioxidant, antimicrobial (with special focus against *A. baumannii*), anti-*quorum sensing*, and anti-inflammatory activities, given that this EO is traditionally used probably because of some of these biological properties.

## 2. Materials and Methods

### 2.1. Essential Oil

*Mentha pulegium* L. EO was extracted from the aerial parts of the shrub (identified by the EO producer), growing spontaneously (organic farming, PT-BIO-02, ECOCERT) in Parque Natural do Vale do Guadiana (Serra de Mértola, Alentejo, Portugal) (voucher specimen deposited in ISA-UL herbarium). The shrub was collected manually and immediately subjected to steam-distillation using a stainless-steel alembic by Herdade Vale Côvo (https://www.herdade-valecovo.com/en/produto/pennyroyal-essential-oil/, accessed on 28 July 2021). The pennyroyal EO was kept in the dark at −20 °C.

### 2.2. Essential Oil Chemical Composition

The components of pennyroyal EO were identified by gas chromatography (GC) coupled to mass spectrometry (MS), being processed in total ion chromatogram (TIC) mode, and quantified by GC with flame ionization detection (FID), using the relative area percentage, according to the standard protocol ISO 11024. An Agilent 7820A GC-FID (Santa Clara, CA, USA) equipped with an Agilent 5977B MS (Santa Clara, CA, USA) detector, along with a DB-WAX ultra-inert GC column (Agilent, Santa Clara, CA, USA) (60 m × 0.25 mm × 0.5 µm), were used. The carrier gas was helium at a head pressure of 33 Psi (FID) and 25.5 Psi (MS), with an injection volume of 0.1 µL for both FID and MS, using the split mode in the mass range from 22 to 350. The oven temperature was programmed for 6 min at 50 °C, 2 °C/min to 190 °C, 4 °C/min to 220 °C, 10 min at 220 °C, 4 °C/min to 250 °C, and finally 10 min at 250 °C [11].

### 2.3. Antioxidant Activity Evaluation

#### 2.3.1. DPPH Free Radical Scavenging Assay

Initially, 3.9 mL of three methanolic solutions (0.2, 0.12, and 0.08 mM) of DPPH (2,2-diphenyl-1-picrylhydrazyl) (Sigma-Aldrich, St. Louis, MO, USA) were mixed with 100 µL of several concentrations of pennyroyal EO (from 5 to 100%, *v/v*). The negative control consisted in a mixture of 3.9 mL of each DPPH solution and 100 µL of methanol. The positive control was prepared with gallic acid (Sigma-Aldrich, St. Louis, MO, USA). These mixtures remained in the dark at room temperature for 90 min. After that time, their absorbances were measured at 517 nm using a spectrophotometer (Helios-Omega, Thermo Scientific, Waltham, MA, USA), considering methanol as blank.

The DPPH scavenging activity was determined as follows [11,12]:

%Inhibition = [(A_control_ − A_sample_)/A_control_] × 100 (1)

where A_control_ corresponds to the absorbance of the negative control and A_sample_ corresponds to the absorbance of the mixtures with pennyroyal EO.

A curve by plotting the pennyroyal EO concentrations *versus* the corresponding % inhibition was used to determine the IC_50_, the value of which was further used to determine the antioxidant activity index (AAI) of the pennyroyal EO [11,12]:

AAI = final concentration of DPPH in the negative control/IC_50_ (2)

The AAI enabled the classification of the pennyroyal EO antioxidant activity as follows: poor (AAI ≤ 0.5), moderate (0.5 < AAI ≤ 1.0), strong (1.0 < AAI < 2.0), or very strong (AAI ≥ 2.0) [11,12]. The DPPH solutions were prepared daily, with this assay being carried out in triplicate.

#### 2.3.2. β-Carotene/Linoleic Acid System

Initially, 40 μL of linoleic acid (TCI Europe N.V., Zwijndrecht, Belgium), 400 μL of Tween 40 (Riedel-de Häen, Seelze, Germany), and 1 mL of chloroform (Scharlab, Barcelona, Spain) were mixed with 500 μL of a β-carotene (Sigma-Aldrich, St. Louis, MO, USA) solution (20 mg/mL in chloroform). Then, the chloroform was removed by evaporation under vacuum, and finally an emulsion was obtained by adding 100 mL of cold distilled water saturated with oxygen to the mixture. Subsequently, 300 μL of several concentrations of pennyroyal EO (from 5 to 100%, *v/v*) were added to 5 mL of the emulsion previously prepared. The negative control was composed by 300 μL of methanol and 5 mL of the emulsion. The positive control was prepared with the synthetic antioxidant butylated hydroxytoluene (BHT) (Sigma-Aldrich, St. Louis, MO, USA). Lastly, the mixtures were vigorously stirred and placed in a water bath at 50 °C for 1 h. After the incubation, their absorbances were measured at 470 nm using a spectrophotometer (Helios-Omega, Thermo Scientific, Waltham, MA, USA), considering an emulsion without β-carotene as blank.

This assay was performed in triplicate, with the antioxidant activity of the pennyroyal EO being determined by Equation (3) [11]:

%Inhibition = [(A^t=1h^_sample_ − A^t=1h^_control_)/(A^t=0h^_control_ − A^t=1h^_control_)] × 100 (3)

where A^t=1h^ is the absorbance of the sample or negative control at the final time of incubation (t = 1 h), and A^t=0h^ is the absorbance of the negative control at the initial time of incubation (t = 0 h).

### 2.4. Antimicrobial Activity Evaluation

#### 2.4.1. Microorganisms and Culture Conditions

The antimicrobial studies were carried out against eleven microbial reference strains purchased in Frilabo (Maia, Portugal): five Gram-negative bacterial strains (*Salmonella* Typhimurium ATCC 13311, *Escherichia coli* ATCC 25922, *Pseudomonas aeruginosa* ATCC 27853, *Acinetobacter baumannii* LMG 1041, and *Acinetobacter baumannii* LMG 1025); four Gram-positive bacterial strains (*Bacillus cereus* ATCC 11778, *Enterococcus faecalis* ATCC 29212, *Staphylococcus aureus* ATCC 25923, and *Listeria monocytogenes* LMG 16779); and two yeast strains (*Candida tropicalis* ATCC 750 and *Candida albicans* ATCC 90028).

Stock cultures were prepared with 20% (*v/v*) glycerol (Merck, Darmstadt, Germany) and stored at −80 °C. The strains were sub-cultured on an appropriate agar plate 24 h before the antimicrobial studies. Sabouraud dextrose agar (SDA) (Liofilchem, Roseto degli Abruzzi, Italy) was used for the growth of yeasts and brain heart infusion agar (BHI) (Liofilchem, Roseto degli Abruzzi, Italy) was used as culture medium for bacteria [13].

#### 2.4.2. Disk Diffusion Assay

The disk diffusion assay was used to evaluate the antimicrobial activity of pennyroyal EO, following the standard protocols described by Clinical Laboratory and Standards Institute (CLSI) (M2-A8 for bacteria and M44-A2 for yeasts). The inocula were prepared by suspending bacteria or fungi in sterile saline solution (NaCl, 0.85% *w/v*) to a cell density of 0.5 McFarland (1 to 2 × 10^8^ colony-forming units/mL (CFUs/mL) to non-fastidious bacteria and 1 to 5 × 10^6^ CFUs/mL for yeasts) measured using a McFarland densitometer (DEN-1, BIOSAN, Riga, Latvia). Sterile blank filter disks with a diameter of 6 mm (Filtres Fioroni, Ingré, France) were impregnated with 15 μL of pennyroyal EO and were then placed on the inoculated agar plates. Negative controls were prepared using 15 µL of dimethyl sulfoxide (DMSO) (Sigma-Aldrich, St. Louis, MO, USA) and positive controls were prepared using tetracycline (Sigma-Aldrich, St. Louis, MO, USA) (30 µg/disk) in the case of bacteria and amphotericin B (Sigma-Aldrich, St. Louis, MO, USA) (25 µg/disk) for yeasts. The agar plates inoculated with bacteria were incubated at 37 °C for 24 h and for 48 h in the case of yeasts. After the incubation period, all the plates were visually examined for inhibition zones and their diameters were measured with a digital pachymeter, including the disk diameter [11,13,14,15]. 

#### 2.4.3. Resazurin Microtiter Method

The values of minimum inhibitory and minimum lethal concentrations (MIC and MLC, respectively) of pennyroyal EO were determined using the resazurin microtiter assay. For bacterial strains, Müeller–Hinton Broth (MHB) (Liofilchem, Roseto degli Abruzzi, Italy) was used as culture medium, with two-fold serial dilutions of the pennyroyal EO (from 0.25 to 32%, *v/v*) being prepared in a 96-well plate (50 μL/well) using a maximum DMSO final concentration of 2% (*v/v*) to improve the solubility of the EO. Subsequently, 10 µL of resazurin indicator solution (TCI Europe N.V., Zwijndrecht, Belgium) (0.1% *w/v* dissolved in MHB) were added to the wells, followed by 30 μL of fresh MHB. Then, 10 µL of the bacterial suspension (0.5 McFarland) were also added to the wells. Several controls were considered in the plates: a positive control composed of a broad-spectrum antibiotic, a column with all solutions except the bacterial suspension, adding the respective volume of MHB instead, a column with the DMSO control, and a column with all solutions except the test compounds. The plates were incubated for 24 h at 37 °C, with this assay being performed at three independent times. Color changes from purple to pink/colorless were visually assessed and recorded [6,16].

The inocula of yeast strains were prepared suspending several colonies in sterile saline solution and adjusting the turbidity of the suspensions to 0.5 McFarland. Resazurin was also used as cell growth indicator, mixing 50 µL of resazurin sterilized solution (20 mg/mL in water) with the working suspension (inoculum diluted 1:1000 in culture medium). The culture medium used for yeasts was prepared by dissolution of 5.125 g of RMPI-1640 (Sigma-Aldrich, St. Louis, MO, USA) supplemented with phenol red and glutamine, without bicarbonate and 35.53 g of 3-(*N*-morpholino)propanesulfonic acid (MOPS) (Sigma-Aldrich, St. Louis, MO, USA) in 400 mL of distilled water. Furthermore, 1 M sodium hydroxide (Scharlab, Barcelona, Spain) was used to adjust the pH to 6.9–7.1 at 25 °C. After that, the final volume was adjusted to 500 mL, with the medium being filter-sterilized and stored and 4 °C until further used. As described above for bacteria, the microdilution susceptibility method was used, changing the final volume in the wells of the microplates to 200 µL. This assay was also performed at three independent times, with the plates incubated for 24 h at 37 °C [17]. The MIC value was taken at the lowest concentration at which color change occurred.

To determine the MLC values, 10 μL were plated from the wells without visible growth, with the number of colonies being counted after incubation. The MLC was defined as the lowest concentration of pennyroyal EO causing 99.9% death of the microbial inoculum.

### 2.5. Anti-Quorum Sensing Activity Evaluation

#### 2.5.1. Biomonitor Strain

*Chromobacterium violaceum* ATCC 12472 was used as a biomonitor strain to evaluate the anti-*quorum sensing* activity of the pennyroyal EO. An overnight aerobic growth culture (30 °C, 250 rpm) in Luria–Bertani (LB) broth (Liofilchem, Roseto degli Abruzzi, Italy) was used to obtain the bacterial suspension of *C. violaceum* ATCC 12472 [18].

#### 2.5.2. Disk Diffusion Assay

*C. violaceum* ATCC 12472 suspension was adjusted to an OD_620 nm_ of 1 and then seeded in LB agar (Liofilchem, Roseto degli Abruzzi, Italy) plates. Sterile disks (6 mm diameter) saturated with 15 µL of pennyroyal EO were placed over the inoculated plates and incubated (30 °C, 24 h). After the incubation period, the inhibition of the violacein pigment production around the disks (a ring of colorless but viable cells) was measured using a digital pachymeter. DMSO was employed as negative control and resveratrol was used as a known *quorum sensing* inhibitor [18].

Additionally, the plates were observed by optical microscopy using a Nikon Labophot-2 microscope (Nikon, Tokyo, Japan) equipped with a Leica MC190 HD camera (Leica, Wetzlar, Germany) and controlled by the LAS v4.13 software (https://imillermicroscopes.com/pages/software-download, accessed on 31 January 2021), in order to verify the violacein production inhibition [15]. These experiments were performed in triplicate.

### 2.6. Anti-Inflammatory Activity Evaluation

As the denaturation of proteins is among the main causes of inflammation, the capacity of the pennyroyal EO to inhibit protein denaturation was evaluated using the previously optimized protocol [6]. A solution of 1% (w/v) of bovine serum albumin (BSA) (Sigma-Aldrich, St. Louis, MO, USA) was prepared in phosphate buffer saline (PBS), with glacial acetic acid (Scharlab, Barcelona, Spain) being used to adjust the pH to 6.8. Subsequently, 900 μL of the BSA solution were mixed with 100 μL of several dilutions of the pennyroyal EO (from 5 to 100%, *v/v*) prepared with DMSO. Acetylsalicilyc acid (Sigma-Aldrich, St. Louis, MO, USA) dissolved in water was used as positive control. Distilled water was considered as the negative control. Initially, the test tubes were preheated for 10 mint at 37 °C, then incubated for 10 min at 72 °C, and finally cooled with ice for another 10 min. The absorbances of the solutions were measured at 620 nm using a spectrophotometer (Helios-Omega, Thermo Scientific, Waltham, MA, USA). The percentage of the inhibition of protein denaturation was calculated as follows [6]:

%Inhibition = 100 − [(A_sample_ × 100)/A_control_] (4)

where A_sample_ is the absorbance of the sample and A_control_ corresponds to the absorbance of the negative control. The results were expressed as IC_50_ values, determined using a previously constructed calibration curve and considering the triplicate values.

### 2.7. Statistical Analysis

Generally, the results were shown as mean ± standard deviation (SD). The IBM SPSS Statistics v25 software (https://www.ibm.com/analytics/spss-statistics-software, accessed on 7 December 2020) was used to analyze the raw values, employing the Student’s *t*-test (assuming the normal distribution of the continuous variables). Differences among means were considered to be significant if the *p*-value was <0.05 (a confidence level of 95%).

## 3. Results and Discussion

Pennyroyal EO, of which major compound is known to be pulegone [10], is traditionally used because of some biological properties it presents, which are probably related to its antioxidant, antimicrobial, and anti-inflammatory activities [10]. Therefore, this work aimed to make a screening of the major bioactivities of the pennyroyal EO, in order for it to be possible to correlate the uses in traditional medicine of this EO and its biological potential.

### 3.1. Chemical Composition

**Figure 1:**
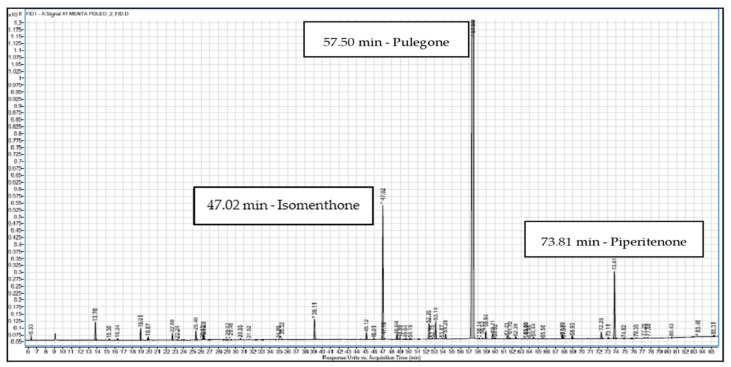
GC-FID chromatogram of the pennyroyal EO.

The study of the volatiles of pennyroyal EO revealed 43 different compounds, accounting for 99.17% of its chemical composition (Table 1). The GC-FID chromatogram obtained is shown in Figure 1. Piperitenone (2.58%), isomenthone (4.60%), and pulegone (86.64%) were the three major compounds, with the other components being present in trace amounts. Pulegone is an oxygenated monoterpene that has recognized antioxidant [19], antimicrobial [20,21], and anti-inflammatory [22] properties. A previous work that studied the chemical composition of a Portuguese pennyroyal EO also reported pulegone as the major compound, with 23.20% of the total EO composition [10]. Such variability in pulegone amount may be related to distinct vegetative phases of the plant when it is harvested, as well as to edaphoclimatic variations.

### 3.2. Antioxidant Activity

Table 2 summarizes the antioxidant activity parameters of the pennyroyal EO, showing that it presented the capacity to scavenge the DPPH free radicals. Despite being significantly lower (*p*-value < 0.05) than that of gallic acid, the antioxidant activity of pennyroyal EO was classified as very strong, according to the scale implemented by Scherer and Godoy, 2009 [12], previously described in Materials and Methods.

Interestingly, the antioxidant activity of the EO assessed by the β-carotene/linoleic acid system, which allows the evaluation of the inhibition of lipid peroxidation, is significantly higher (*p*-value < 0.05) than that of BHT, presenting a significantly lower (*p*-value < 0.05) IC_50_ value. Other researchers reached similar results of antioxidant activity when studying pennyroyal EO simultaneously with methanolic and aqueous extracts of *M. pulegium* with the objective of using the pennyroyal-derived products in the conservation of sunflower oil during storage [19].

Cheraif, et al. (2020) evaluated the antioxidant activity of an Algerian pennyroyal EO, also composed mostly by pulegone (76.9%), and concluded that the EO presented lower capacity to scavenge the DPPH free radicals when compared with ascorbic acid used as control [23].

### 3.3. Antimicrobial Activity

The results of the antimicrobial activity of pennyroyal EO against the different microorganisms tested are summarized in Table 3. It was observed that the highest inhibition zones were obtained against the strains of *A. baumannii* (>30 mm).

The MIC values of the EO (Table 4) for *A. baumannii* were the lowest (2%, *v/v*), confirming its selective action against these strains of Gram-negative bacterium. Furthermore, because the MIC values are equal to those of the MLC, it can be said that pennyroyal EO may present a bactericidal action, as other previous reports suggested [2,4,6]. Although previous studies have already described the potential antimicrobial action of *M. pulegium* EO [20,21], none of these studies have evaluated its activity against *A. baumannii*.

### 3.4. Anti-Quorum Sensing Activity

The anti-*quorum sensing* activity of pennyroyal EO was evaluated using the biomonitor strain *C. violaceum* ATCC 12472, which normally produces the purple compound violacein in response to the presence of *N*-hexanoyl homoserine lactone (autoinducer molecule) [24].

**Figure 2:**
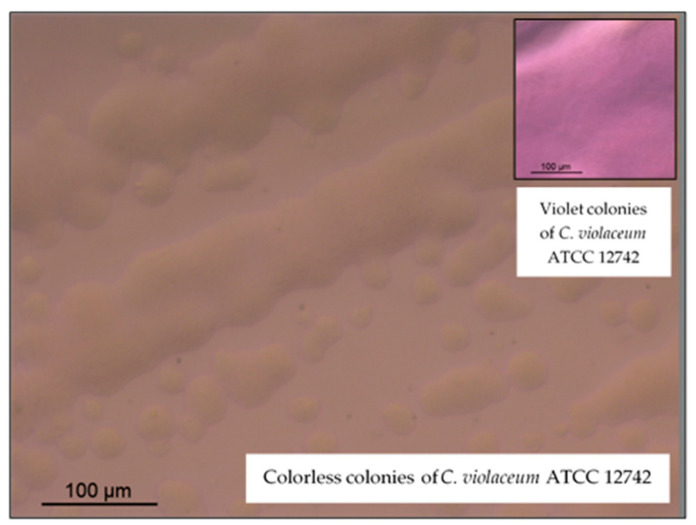
Optical microscopy images showing the inhibition of the violacein pigment production (magnification: 100×).

The EO presented a significantly higher (*p*-value < 0.05) capacity to inhibit the production of violacein than resveratrol (Table 5), indicating its ability to act as *quorum quencher*. This result is of utmost importance, because several virulence mechanisms, including biofilm formation and antimicrobial resistance, can be regulated through *quorum sensing* mechanisms; identifying compounds that have the capacity to inhibit these mechanisms can contribute to reducing virulence and pathogenicity of microorganisms. These results were confirmed by the images of optical microscopy that showed colorless colonies of *C. violaceum* near the disk impregnated with pennyroyal EO, contrariwise to what was observed with DMSO (Figure 2). Zheng, et al. (2020) also reported that *M. pulegium* EOs have quorum sensing-inhibitory activity in *Vibrio campbellii* BB120 [25].

### 3.5. Anti-Inflammatory Activity

Concerning the anti-inflammatory activity of pennyroyal EO, it was possible to verify that the value of IC_50_ obtained was quite similar to the one obtained for acetylsalicylic acid (Table 6). This result suggests the potential anti-inflammatory action of the EO, which is probably owing to the high content of pulegone. It was previously demonstrated that pulegone can inhibit the hypersecretion of IL-1β and IL-18 as well as other inflammation mechanisms [22].

Other researchers also reported that pennyroyal phenolic extract reduces TNBS-induced colitis inflammation markers [26]. Star-anise EO was shown to present a more pronounced anti-inflammatory activity (IC_50_ = 12.03%, *v/v*) measured by the same method [6].

## 4. Conclusions

The results obtained in this work demonstrated that pennyroyal EO, mainly composed by pulegone, has antioxidant, antimicrobial, anti-*quorum sensing*, and anti-inflammatory activities. It is worth highlighting the excellent activity of pennyroyal EO against *Acinetobacter baumannii*, which, associated with its ability to inhibit *quorum sensing*, can be suggested for use in the development of surface disinfectants against this microorganism that has emerged in recent years as the main cause of nosocomial infections.

## Figures and Tables

**Table 1:** Chemical composition of pennyroyal EO assessed by GC analysis.

Retention Time (min)	Area	*m*/*z* Predicted *	Compounds	Relative %
13.76	285,520.68	136.13	α-Pinene	0.50
15.36	13,893.17	100.09	2,5-Diethyltetrahydrofuran	0.02
19.01	209,964.64	136.13	β-Pinene	0.37
19.87	54,199.40	136.13	Sabinene	0.09
22.68	115,020.48	136.13	β-Myrcene	0.20
23.24	9218.27	136.13	*p*-Mentha-1(7),8-diene	0.02
25.40	157,870.05	136.13	Limonene	0.27
26.29	92,843.29	154.14	1,8-Cineole	0.16
29.07	5981.18	136.13	γ-Terpinene	0.01
29.46	5959.16	128.12	3-Octanone	0.01
30.55	15,695.05	164.34	*p*-Cymene	0.03
31.52	6537.28	136.13	α-Terpinolene	0.01
34.99	13,341.94	112.09	3-Methyl-Cyclohexanone	0.02
35.32	14,539.98	172.15	Octan-3-yl Acetate	0.03
39.11	432,642.07	130.14	3-Octanol	0.75
45.12	131,858.94	154.14	Menthone	0.23
46.01	12,943.67	150.11	Menthofuran	0.02
47.02	2,646,297.16	154.14	Isomenthone	4.60
48.64	95,801.61	152.12	Camphor	0.17
48.99	13,603.15	204.19	β-Bourbonene	0.02
49.64	9923.48	154.14	Linalool	0.02
50.19	9195.44	204.19	β-Cubebene	0.02
52.35	309,997.70	152.12	*cis*-Isopulegone	0.54
53.14	359,242.06	152.12	*trans*-Isopulegone	0.62
53.87	20,479.81	154.14	Terpinen-4-ol	0.04
54.28	108,658.39	204.19 + 168.15	*trans*-β-Caryophyllene + *p*-Menth-3-en-8-ol	0.19
57.50	49,804,964.30	152.12	Pulegone	86.64
58.94	160,304.10	204.19	α-Humulene	0.28
59.71	100,746.80	154.14	α-Terpineol	0.18
60.02	18,054.61	202.17	Dehydro-Aromadendrene	0.03
61.43	68,102.24	204.19	*D*-Germacrene	0.12
61.72	24,673.92	168.12	*cis*-Piperitone Epoxide	0.04
62.39	40,006.84	152.12	Piperitone	0.07
63.60	6533.06	156.15	Citronellol	0.01
63.99	11,083.13	204.19	Δ-Cadinene	0.02
65.56	6698.69	152.12	Myrtenol	0.01
67.78	74505.24	148.09	Anethole	0.13
67.90	12,588.66	168.12	*trans*-Piperitone Epoxide	0.02
73.81	1,485,580.23	150.11	Piperitenone	2.58
76.35	16,187.02	148.09	Shisofuran	0.03
83.46	5906.44	150.11	Thymol Isomer	0.01
85.31	21,831.71	150.11	Carvacrol	0.04
Total identified			43 compounds	99.17

* The *m*/*z* obtained were considered with a mass error of 1 ppm, corresponding to an uncertainty in *m*/*z* of 0.000136.

**Table 2:** Antioxidant properties of pennyroyal EO.

Method	Parameters	Pennyroyal EO ^a^	Gallic Acid ^b^	BHT ^c^	*p*-Values
DPPH	IC_50_ (%, *v/v*)	1.29 ± 0.10	0.25 ± 0.02	-	0.002 ^ab,^*
	AAI	4.02 ± 0.57	23.47 ± 0.34	-	<0.001 ^ab,^*
	Antioxidant Activity	Very Strong	Very Strong	-	-
β-Carotene/Linoleic Acid	IC_50_ (%, *v/v*)	1.15 ± 0.01	-	7.70 ± 0.62	0.003 ^ac,^*

Results expressed as mean ± SD; AAI—antioxidant activity index; BHT—butylated hydroxytoluene; upper letters (a, b, c) were used to identify the pairs of samples under statistical comparison; * indicates a significant result (*p*-value < 0.05).

**Table 3:** Diameters of inhibition zones (mm), including the disk diameter (6 mm).

Microorganisms	Pennyroyal EO (15 µL/disk) ^a^	Tetracycline (30 µg/disk) ^b^	Amphotericin B (25 µg/disk) ^c^	*p*-Values
*S. aureus* ATCC 25923	17.01 ± 1.22	30.32 ± 0.51	-	<0.001 ^ab,^*
*L. monocytogenes* LMG 16779	18.76 ± 0.47	18.31 ± 0.67	-	0.401 ^ab^
*E. faecalis* ATCC 29212	10.73 ± 0.15	25.21 ± 0.65	-	<0.001 ^ab,^*
*B. cereus* ATCC 11778	19.53 ± 1.64	30.01 ± 0.80	-	0.002 ^ab,^*
*E. coli* ATCC 25922	17.06 ± 0.76	23.36 ± 0.56	-	0.001 ^ab,^*
*A. baumannii* LMG 1025	34.84 ± 0.66	25.67 ± 0.33	-	<0.001 ^ab,^*
*A. baumannii* LMG 1041	37.75 ± 2.18	27.48 ± 0.29	-	0.013 ^ab,^*
*S.* Typhimurium ATCC 13311	12.54 ± 1.22	28.53 ± 0.49	-	0.001 ^ab,^*
*P. aeruginosa* ATCC 27853	8.02 ± 1.07	11.59 ± 0.64	-	0.013 ^ab,^*
*C. albicans* ATCC 90028	17.17 ± 0.86	-	20.31 ± 0.58	0.009 ^ac,^*
*C. tropicalis* ATCC 750	18.45 ± 0.74	-	21.49 ± 0.47	0.006 ^ac,^*

Results expressed as mean ± SD; upper letters (a, b, c) were used to identify the pairs of samples under statistical comparison; * indicates a significant result (*p*-value < 0.05); DMSO (15 µL/disk) was used as negative control.

**Table 4:** Minimum inhibitory concentrations (MICs) and minimum lethal concentrations (MLCs) of pennyroyal EO.

Microorganisms	Pennyroyal EO (%, *v/v*) ^a^	Tetracycline (µg/mL) ^b^	Amphotericin B (µg/mL) ^c^	*p*-Values
*S. aureus* ATCC 25923	8	0.06	-	<0.001 ^ab,^* <0.001 ^ac,^*
*L. monocytogenes* LMG 16779	8	0.06	-	<0.001 ^ab,^* <0.001 ^ac,^*
*E. faecalis* ATCC 29212	4	0.06	-	<0.001 ^ab,^* <0.001 ^ac,^*
*B. cereus* ATCC 11778	8	0.06	-	<0.001 ^ab,^* <0.001 ^ac,^*
*E. coli* ATCC 25922	8	0.06	-	<0.001 ^ab,^* <0.001 ^ac,^*
*A. baumannii* LMG 1025	2	0.06	-	<0.001 ^ab,^* <0.001 ^ac,^*
*A. baumannii* LMG 1041	2	0.06	-	<0.001 ^ab,^* <0.001 ^ac,^*
*S.* Typhimurium ATCC 13311	32	0.24	-	<0.001 ^ab,^* <0.001 ^ac,^*
*P. aeruginosa* ATCC 27853	16	0.24	-	<0.001 ^ab,^* <0.001 ^ac,^*
*C. albicans* ATCC 90028	16	-	0.24	<0.001 ^ab,^* <0.001 ^ac,^*
*C. tropicalis* ATCC 750	16	-	0.48	<0.001 ^ab,^* <0.001 ^ac,^*

Results expressed as modal values; the MIC and MLC values are the same; upper letters (a, b, c) were used to identify the pairs of samples under statistical comparison; * indicates a significant result (*p*-value < 0.05).

**Table 5:** Anti-quorum-sensing activity of pennyroyal EO.

Samples	Diameters of Inhibition of the Violacein Pigment Production (mm)
Pennyroyal EO (15 µL/disk) ^a^	89.97 ± 1.22
DMSO (15 µL/disk) ^b^	0.00
Resveratrol (5 µg/disk) ^c^	8.59 ± 0.25
*p*-Values	<0.001 ^ab,^* <0.001 ^ac,^*

Results expressed as mean ± SD; upper letters (a, b, c) were used to identify the pairs of samples under statistical comparison; * indicates a significant result (*p*-value < 0.05).

**Table 6:** Anti-inflammatory activity of pennyroyal EO.

Samples	Anti-Inflammatory Activity—IC_50_ (%, *v/v*)
Pennyroyal EO ^a^	88.31 ± 1.37
DMSO ^b^	>100
Acetylsalicylic acid ^c^	89.47 ± 2.64
*p*-Values	0.005 ^ab,^* 0.548 ^ac^

Results expressed as mean ± SD; upper letters (a, b, c) were used to identify the pairs of samples under statistical comparison; * indicates a significant result (*p*-value < 0.05).

## Data Availability

The data presented in this study are available on request from the corresponding author.
